# Tick saliva protein Evasin-3 modulates chemotaxis by disrupting CXCL8 interactions with glycosaminoglycans and CXCR2

**DOI:** 10.1074/jbc.RA119.008902

**Published:** 2019-06-24

**Authors:** Stepan S. Denisov, Johannes H. Ippel, Alexandra C. A. Heinzmann, Rory R. Koenen, Almudena Ortega-Gomez, Oliver Soehnlein, Tilman M. Hackeng, Ingrid Dijkgraaf

**Affiliations:** ‡ Department of Biochemistry, University of Maastricht, Cardiovascular Research Institute Maastricht, 6229 ER, Maastricht, The Netherlands; § Institute for Cardiovascular Prevention, Ludwig Maximilian University, 80336, Munich, Germany; ¶ German Center for Cardiovascular Research, 13316, Berlin, Germany; ‖ Partner Site Munich Heart Alliance, 80802 Munich, Germany; ** Department of Physiology and Pharmacology and Department of Medicine, Karolinska Institutet, SE-171 77 Stockholm, Sweden

**Keywords:** chemokine, peptide chemical synthesis, nuclear magnetic resonance (NMR), protein structure, protein-protein interaction, C-X-C motif chemokine ligand (CXCL)

## Abstract

Chemokines are a group of chemotaxis proteins that regulate cell trafficking and play important roles in immune responses and inflammation. Ticks are blood-sucking parasites that secrete numerous immune-modulatory agents in their saliva to evade host immune responses. Evasin-3 is a small salivary protein that belongs to a class of chemokine-binding proteins isolated from the brown dog tick, *Rhipicephalus sanguineus*. Evasin-3 has been shown to have a high affinity for chemokines CXCL1 and CXCL8 and to diminish inflammation in mice. In the present study, solution NMR spectroscopy was used to investigate the structure of Evasin-3 and its CXCL8–Evasin-3 complex. Evasin-3 is found to disrupt the glycosaminoglycan-binding site of CXCL8 and inhibit the interaction of CXCL8 with CXCR2. Structural data were used to design two novel CXCL8-binding peptides. The linear tEv3 17–56 and cyclic tcEv3 16–56 dPG Evasin-3 variants were chemically synthesized by solid-phase peptide synthesis. The affinity of these newly synthesized variants to CXCL8 was measured by surface plasmon resonance biosensor analysis. The *K_d_* values of tEv3 17–56 and tcEv3 16–56 dPG were 27 and 13 nm, respectively. Both compounds effectively inhibited CXCL8-induced migration of polymorphonuclear neutrophils. The present results suggest utility of synthetic Evasin-3 variants as scaffolds for designing and fine-tuning new chemokine-binding agents that suppress immune responses and inflammation.

## Introduction

Chemokines are a diverse group of chemotaxis proteins that guide cell trafficking and play an important role in diverse physiological processes such as immune response, inflammation, angiogenesis, and cell differentiation (1). Although chemokines structurally fall into two major (CC and CXC) and two minor (XC and CX3C) groups depending on their cysteine motif, all chemokines share the similar spatial topology. Common structural features include a flexible N terminus, N-loop, and single-turn 3_10_-helix followed by three β-strands and a C-terminal α-helix (1). Three β-strands are connected to each other by 30s and 40s loops, whereas the 50s loop links the C-terminal α-helix to the β_3_-strand. Chemokines control cell trafficking by interactions with chemokine G protein–coupled receptors (GPCRs)^2^ and endothelial cells glycosaminoglycans (GAGs). Chemokines activate GPCRs through binding of the receptor N terminus (chemokine recognition site 1) and the transmembrane pocket (chemokine recognition site 2) via its globular core (the N-loop and 40s loop) and flexible N terminus, respectively (2–4). Chemokine–receptor interactions are redundant, and usually several chemokines can activate one GPCR (5). At the same time, low-affinity interactions of chemokines with GAGs create haptotactic gradients that direct cell migration (6). Combined with chemokine oligomerization (7) and heterodimerization (8, 9), these interactions form a complex signaling network that regulates immune and inflammatory responses.

Pathogens such as viruses, worms, and ticks target different parts of the chemokine network to avoid the host immune response (10–12). Ticks gain particular interest because their saliva contains numerous bioactive compounds, including proteins, and could be a rich source of candidates for drug development (13). Among those bioactive compounds are Evasins, a class of chemokine-binding proteins first isolated from saliva of the brown dog tick *Rhipicephalus sanguineus* (14, 15). Until now, the class consisted of three family members: Evasin-1, Evasin-3, and Evasin-4. However, several hundreds of proteins distantly related to Evasins have been identified in eight different tick species (16). In general, Evasins can be divided up in two subclasses: C8 fold (Evasin-1 and Evasin-4) and C6 fold (Evasin-3) containing 8 and 6 cysteines, respectively. Evasin-1 and Evasin-4 show efficient binding to human CC-chemokines, such as CCL5 (RANTES) and CCL3, whereas Evasin-3 shows high affinity to CXC-type chemokines, in particular CXCL8 (interleukin-8) and CXCL1 (Gro-α).

CXCL8 is an inflammatory chemokine associated with the development of numerous diseases such as neurodegenerative and pulmonary disorders, several types of cancer, psoriasism, and rheumatism (17–19). It has been shown that direct targeting of CXCL8 by antibodies has beneficial effects during acute lung injury (20), meconium aspiration syndrome (21), chronic obstructive pulmonary disease (22), and psoriasis (23). Several low-affinity CXCL8-binding peptides and peptidomimetics have been derived from CXCR1/2 chemokine-binding sites and showed activity in both *in vitro* and *in vivo* studies (24, 25). Administration of Evasin-3 has a beneficial effect during myocardial ischemia, which is attributed to its chemokine-binding activity (26). Thus, Evasin-3 could be considered as a promising alternative to antibodies and chemokine-binding peptides, combining high-affinity and easy accessibility by chemical synthesis because of its relatively small size.

In contrast to Evasin-1 (27) and Evasin-4 (28), Evasin-3 has not been in the focus of structural research, and no structures of Evasin-3 or its complexes with chemokines are available so far. In the present study, we investigated structures of Evasin-3 and the CXCL8–Evasin-3 complex using solution NMR spectroscopy. Furthermore, we developed two new high-affinity CXCL8-binding peptides based on the experimentally determined structure of the CXCL8–Evasin-3 complex. Both peptides showed potent anti-inflammatory activity *in vitro*.

## Results

### Expression of Evasin-3

Expression of recombinant Evasin-3 in *Escherichia coli* yielded two major products with molecular masses of 7,000 and 7,130 Da that corresponded to native Evasin-3 and a variant with a N-terminal methionine (designated as met-Evasin-3), respectively (Fig. S1). Oxidative folding in the presence of the cysteine/cystine redox couple resulted in a 6 Da decrease of the molecular mass for both variants, indicating the loss of six protons with formation of three disulfide bonds. The expression level of met-Evasin-3 was substantially higher, and for that reason this variant was used for further NMR spectroscopy studies.

### NMR analysis of [^15^N,^13^C] met-Evasin-3

**Figure 1.**
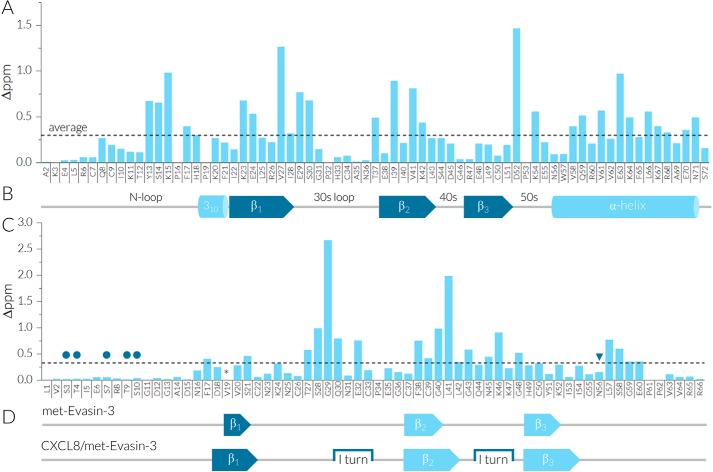
**NMR analysis of binding CXCL8 to met-Evasin-3.** *A*, the chemical shift perturbation plot of 200 μm [^15^N,^13^C]CXCL8 amide peaks upon binding of 200 μm met-Evasin-3 at 37 °C, pH 4.5. *B*, schematic representation of CXCL8 secondary structure according to the dimer crystal structure (PDB code 1IL8). *C*, the chemical shift perturbation plot of 200 μm [^15^N,^13^C] met-Evasin-3 amide peaks upon binding of 200 μm CXCL8 at 44 °C, pH 4.5. Predicted *O-*glycosylation sites are marked by *circles*, and *N*-glycosylation is shown by *triangles. D*, schematic representation of met-Evasin-3 secondary structure predicted by CSI 3.0 in the unbound form (*top*) and in the complex with CXCL8 (*bottom*). Edge β-strands are shown by *dark blue arrows*, interior β-strands are shown by *light blue arrows*, and turns are shown by *square brackets*. Δppm values are expressed as the sum of square roots (Δ^15^N_free–complex_)^2^/6.51 + (Δ^1^H_free–complex_)^2^.

**Figure 2.**
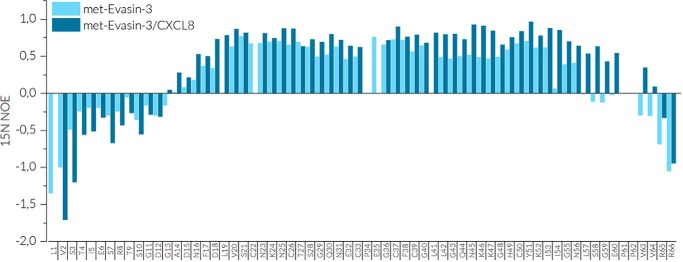
**^15^N heteronuclear NOE relaxation values of [^15^N,^13^C]met-Evasin-3 in free form (*light blue*) and in the CXCL8–[^15^N,^13^C]met-Evasin-3 complex (*dark blue*).**

Sequential assignment of met-Evasin-3 was carried out by a combination of 2D and triple resonance 3D spectra. Analysis of ^15^N-^1^H HSQC spectra at various temperatures and concentrations showed the presence of extensive line broadening of proton signals, which could only be resolved by recording spectra at elevated temperature (44 °C). Under these conditions all expected amide peaks in met-Evasin-3 were observed with the exception of Val^19^ (Fig. S2*A*). In addition, conformational heterogeneity of certain amide peaks was observed for the Ser^3^–Arg^8^ and Ser^58^–Arg^66^ regions that relates to intermolecular aggregation at higher protein concentrations. The met-Evasin-3 secondary structure was first predicted by the CSI 3.0 webserver using available experimental chemical shifts for backbone atoms. According to these chemical shifts, met-Evasin-3 consists of three short β-strands in Val^19^–Ser^21^, Cys^37^–Gly^40^, and His^49^–Lys^52^ regions, whereas the rest of the protein is predicted to be in the random coil state (Fig. 1*D*). Heteronuclear ^15^N NOE relaxation values were measured to determine flexibility disorder of met-Evasin-3 (Fig. 2). Negative values for residues in the N-terminal Leu^1^–Asp^15^ and C-terminal Leu^57^–Arg^66^ regions indicated high backbone flexibility, whereas the cysteine-rich core Ser^21^–Tyr^51^ showed positive relaxation values >0.5 corresponding to a relatively rigid structure.

### NMR analysis of CXCL8–met-Evasin-3 complex

The complex of [^15^N,^13^C]CXCL8 with unlabeled met-Evasin-3 and the reverse isotope-labeled system was used to study the structure of the CXCL8–met-Evasin-3 complex by NMR spectroscopy. Titration of [^15^N,^13^C]CXCL8 by unlabeled met-Evasin-3 caused changes of chemical shifts of CXCL8 amide atoms, although complex formation proceeded relatively slowly at pH 4.5, and final equilibrium was only achieved after incubation up to 1 h at 37 °C (Fig. S3). After reaching equilibrium at a molar ratio of 1:1, further addition of met-Evasin-3 did not lead to changes in the ^15^N-^1^H HSQC spectrum, indicating 1:1 stoichiometry of the CXCL8–met-Evasin-3 complex. The pattern of met-Evasin-3–induced chemical shift changes of amide atoms in CXCL8 demonstrated that most affected residues were located in the β1- and β2-strands and the α-helix of CXCL8 (Fig. 1, *A* and *B*). Significant changes in chemical shifts were also observed for the N-loop (Tyr^13^–Lys^15^) and the 50s loop (Asp^52^–Lys^54^).

**Figure 3.**
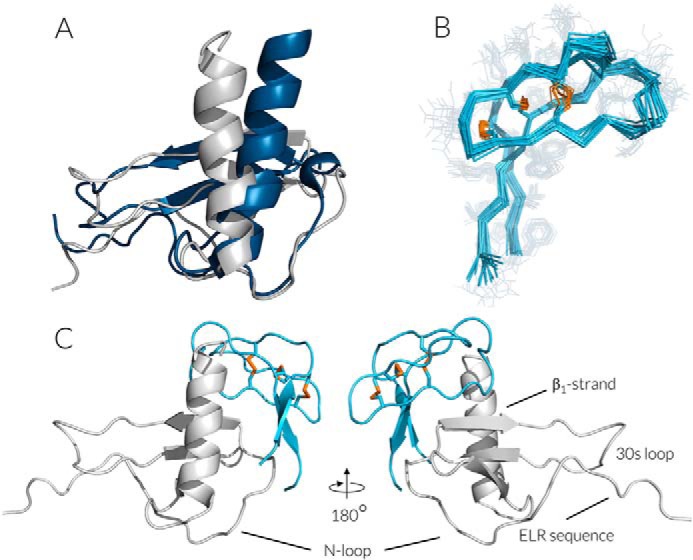
*A*, overlay of CXCL8 structures in the CXCL8 dimer (*dark blue*; PDB code 1IL8) and in the [^15^N,^13^C]CXCL8–met-Evasin-3 complex (*gray*). *B*, ribbon representation of the ensemble of 10 lowest energy structures of tEv3 17–56 (PDB code 6QJB); disulfides are shown by *yellow sticks. C*, cartoon representation of the HADDOCK structure of the CXCL8–tEv3 17–56 complex. tEv3 17–56 is shown in *light blue*, and CXCL8 is in *gray*.

At NMR concentrations (1–500 μm), CXCL8 is present as a dimer. The absence of CXCL8 dimer-specific intermolecular NOE signals, *e.g.* Glu^29^–Ala^69^, Glu^29^–Leu^25^, in the [^15^N,^13^C]CXCL8–met-Evasin-3 complex indicates that binding of met-Evasin-3 caused CXCL8 dimer disruption (Fig. S4). NOE restraints extracted from ^13^C- and ^15^N-edited NOESY spectra and TALOS-predicted dihedral angles were used to calculate the set of CXCL8 structures in the complex with met-Evasin-3 (Fig. S5, *A* and *B*). In contrast to the CXCL8 structure in the dimer, the CXCL8 α-helix in the CXCL8–met-Evasin-3 complex is repositioned and oriented almost orthogonally to the β-sheet (Fig. 3*A*). The region Phe^17^–Glu^24^ is twisted and pushed away forming the cavity between the CXCL8 N-loop and the α-helix.

Similarly, NMR spectra were recorded for the reverse-labeled CXCL8–[^15^N,^13^C]met-Evasin-3 complex. Binding of CXCL8 to met-Evasin-3 drastically decreased line broadening of the most core met-Evasin-3 NMR resonances. The CXCL8-induced chemical shift perturbation pattern of amide peaks is displayed in Fig. 1, *C* and *D*. Spectral changes were especially high for the Thr^27^–Gln^30^ and Cys^38^–Leu^41^ regions. Heteronuclear NOE relaxation data indicated that binding of CXCL8 increased rigidity of the met-Evasin-3 C-terminal Asn^56^–Val^63^ and Leu^42^–Lys^47^ residues, whereas the N-terminal part Leu^1^–Asp^12^ showed even higher flexibility (Fig. 2). Met-Evasin-3 in the complex possessed more defined secondary structure compared with that of the free form (Fig. 1*B*). All three β-strands were extended upon binding of CXCL8, and two I type turns were also predicted in regions Gln^30^–Cys^33^ and Gln^44^–Lys^47^. The structure of [^15^N,^13^C]met-Evasin-3 in the CXCL8–[^15^N,^13^C]met-Evasin-3 complex was calculated using integrated NOE peaks and TALOS-predicted dihedral angles (Fig. S5, *C* and *D*). Average root-mean-square-deviation values for backbone atoms in the Phe^17^–Asn^56^ region were calculated to be 2.0 Å, and we used these structures mainly as an auxiliary tool for further manual NOESY spectra assignment.

Residues involved in complex formation were mapped using ^15^N- and ^13^C-filtered NOESY experiments on the [^15^N,^13^C]CXCL8–met-Evasin-3 complex. Multiple intermolecular NOE signals were observed between the Phe^38^–Leu^42^ region of met-Evasin-3 and the Leu^25^–Ile^28^ region of CXCL8 in both ^15^N and ^13^C NOESY spectra (Fig. S6). Strong NOE signals were found between Val^61^ in the CXCL8 α-helix and residues Val^19^, Ile^53^, and Phe^38^ of met-Evasin-3.

### GAGs and met-Evasin-3 competition for [^15^N,^13^C]CXCL8 binding

To study competition between Evasin-3 and GAGs for CXCL8 binding, [^15^N,^13^C]CXCL8 was first titrated with increasing concentrations of dalteparin (Fragmin®, Pfizer) and low-molecular-mass heparin with the average molecular mass of 5 kDa. Unfortunately, the addition of Fragmin caused intensity losses and significant deterioration of NMR spectra, most likely caused by aggregation. Fondaparinux (Arixtra®, GSK), which comprises the five central sulfated sugar units in heparin, has the well-defined composition and the lower molecular mass (1.7 kDa) comparing with Fragmin. For that reason, fondaparinux facilitates collection of NMR spectra of higher quality and was chosen for further NMR titration experiments. The highest perturbations by fondaparinux were observed for residues of the CXCL8 α-helix (Trp^57^, Arg^60^, Arg^68^, and Glu^70^) and the N-loop (Lys^15^, His^18^, and Lys^23^) (Fig. S7). Observed changes were consistent with results of previous studies describing the interaction of CXCL8 with several heparin oligosaccharides (29). The addition of met-Evasin-3 to the CXCL8–GAG (at a 1:1 ratio) complex completely abolished GAG binding, and peak positions of CXCL8 slowly reverted back, corresponding to the CXCL8–met-Evasin-3 complex, even in the case of 10-fold excess of fondaparinux (Fig. S8).

### Size-exclusion chromatography of CXCL8–met-Evasin-3 complex

To support findings from previous NMR experiments, CXCL8–met-Evasin-3 complex formation was assessed by size-exclusion chromatography. CXCL8 at a concentration of 0.1 mg/ml eluted into a single unsymmetrical peak that corresponded to the molecular mass of dimeric CXCL8 (Fig. S9*A*). The addition of met-Evasin-3 at a concentration of 0.1 mg/ml caused no immediate changes; however, after a 1-h incubation at 37 °C, the peak shifted to a higher molecular mass of 17 kDa. When obligatory monomeric V27P/E29P CXCL8 (30) was used in the similar experiment, the peak moved immediately from 8 to 17 kDa, indicating significantly faster kinetics of the complex formation (Fig. S9*B*). Observed higher molecular masses of met-Evasin-3 and CXCL8–met-Evasin-3 complex were probably caused by met-Evasin-3 unstructured termini, which would increase the hydrodynamic radius of both free Evasin-3 and the CXCL8–met-Evasin-3 complex.

**Figure 4.**
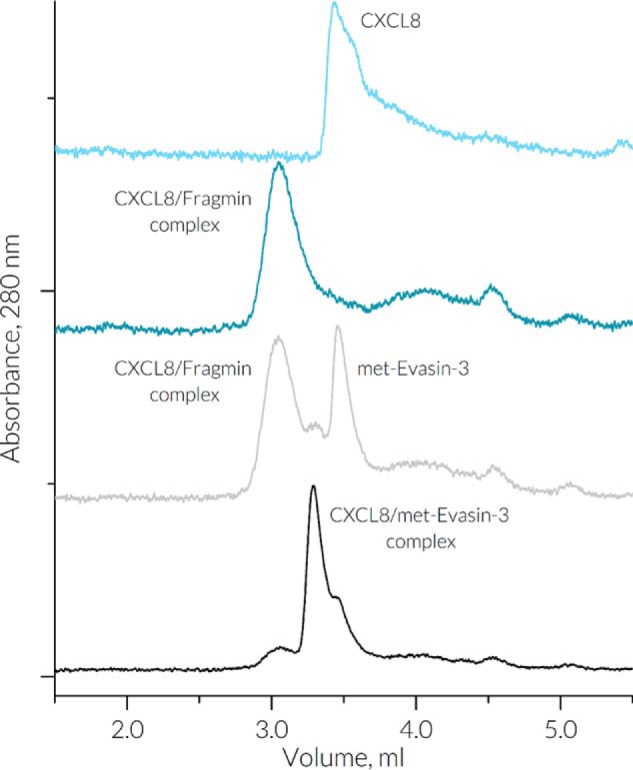
**Chromatographic elution profiles of 0.1 mg/ml CXCL8 in absence (*light blue*) and presence (*dark blue*) of 1 mg/ml of low-molecular-mass heparin (Fragmin).** SEC chromatograms taken immediately after the addition and after a 1-h incubation at 37 °C with 0.1 mg/ml met-Evasin-3 are depicted in *gray* and *black*, respectively.

The mixture of 1 mg/ml low-molecular-mass heparin (Fragmin®) and 0.1 mg/ml of CXCL8 eluted as a single broad peak, indicating formation of the CXCL8–GAG complex with a molecular mass of 35 kDa (Fig. 4). When met-Evasin-3 was added to this CXCL8–GAG complex, the mixture eluted as two separate peaks with molecular masses of 35 and 10 kDa, whereas only the peak corresponding to 17 kDa was observed after a 1-h incubation at 37 °C.

### Truncated Evasin-3 (tEv3 17–56)

To study the relative importance of the flexible unstructured N and C termini of met-Evasin-3 on binding CXCL8, the Leu^1^–Asn^16^ and Leu^57^–Arg^66^ regions were removed, leading to a truncated Evasin-3 variant, designated as tEv3 17–56 (31). Truncation resulted in largely diminished broadening ^1^H NMR resonances, gaining a higher number of NOE peaks necessary for calculation of a better resolved structure. In addition, TALOS-predicted dihedral angles and experimentally assigned disulfide bonds (31) were used to obtain the final high resolution structure of tEv3 17–56 (Fig. 3*B* and Table 1). tEv3 17–56 adopts an L-shaped structure. The short arm of the L is formed by two loops, Thr^27^–Glu^32^ and Gly^40^–Asp^49^, connected together by the Pro^34^–Phe^38^ loop. In contrast to the rigid Thr^27^–Glu^32^ loop, the tip of the Gly^40^–Asp^49^ loop, formed by residues Leu^42^–Asn^45^, is more flexible. Loops are arranged by six cysteines linked into the disulfide pattern where the Cys^26^–Cys^39^ disulfide bond protrudes through the ring formed by the Cys^22^–Cys^37^ and Cys^33^–Cys^50^ disulfides. The flanking Asp^18^–Ser^21^ and His^49^–Ile^54^ regions constitute two short β-strands and form the long arm of the L-shaped structure.

**Table 1:** **Overview of NMR restraints and XPLOR refinement statistics determined for tEv3 17–56**

Experimental constraints	
Long	222
Medium [1 < (*i* − *j*) < 5]	75
Sequential [(*i* − *j*) = 1]	197
Intraresidue [*i* = *j*]	342
Total	836
Dihedral angle constraints	50
Number of restraints per residue	22.2
Number of long-range restraints per residue	5.6
Average atomic root-mean-square deviation to the mean structure (Å)	
Backbone	0.6
Heavy atoms	1.1
Global quality scores (mean/*Z* score)	
Verify3D	0.47/0.16
Prosall	0.48/−0.70
PROCHECK (ϕ–ψ)	−0.33/−0.98
PROCHECK (all)	−0.36/−2.13
MolProbity clash score	1.75/1.23
Ramachandran statistics (% of all residues)	
Most favored	88.2
Additionally allowed	9.5
Generously allowed	2.3
Disallowed regions	0.0

tEv3 17–56 tightly binds CXCL8 at a 1:1 stoichiometry and results in a similar pattern of CXCL8 chemical shifts backbone perturbations as was observed for full-length met-Evasin-3 (Fig. S10). Only Lys^20^ and Lys^64^ residues of CXCL8 do not follow the same tendency and showed substantial difference compared with the met-Evasin-3-induced pattern. Taking into account that tEv3 17–56 binds CXCL8 in the same way as full-length met-Evasin-3, the model of CXCL8–tEv3 17–56 complex was calculated using HADDOCK. Calculated structures of CXCL8 in the CXCL8–met-Evasin-3 complex and tEv3 17–56 were used as a starting point for calculation and combined with experimentally observed NOE contacts in the CXCL8–met-Evasin-3 complex. tEv3 17–56 N and C termini fitted into the cavity between the α-helix and the Phe^17^–Glu^24^ loop, whereas the Gly^40^–Asp^49^ loop binds to the β1-strand of CXCL8 (Fig. 3*C* and Table S1). According to the low-energy models, both the tEv3 17–56 N and C termini were located in proximity of Lys^20^ and Lys^64^ of CXCL8 that could explain the difference in the chemical shift of these CXCL8 residues.

### Truncated cyclic Evasin-3

**Figure 5.**
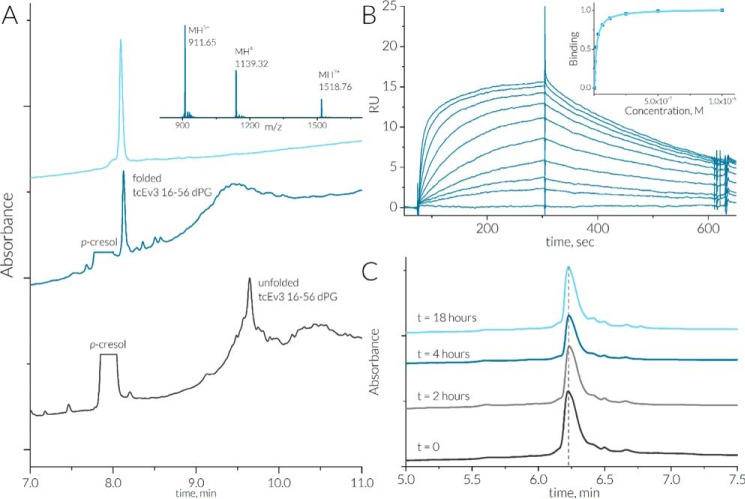
*A*, overview of tcEv3 16–56 dPG folding and purification. The chromatograms of crude peptide mixture after HF cleavage and after folding are shown in *black* and *dark blue*, respectively. The results of the LC-MS analysis of purified tcEv3 16–56 dPG is shown in *light blue*; the calculated mass of [MH]^+^ is 4552.02 Da, and the observed mass is 4552.06 Da. *B*, SPR biosensor analysis of tcEv3 16–56 dPG upon binding to immobilized human CXCL8. The *binding curve* is plotted using maximal response signal for each injection. The apparent *K_d_* value is calculated by fitting the data to a steady-state affinity model using a linear component. The fitted *K_d_* is 13 nm, and *R*_max_ = 16.4, χ^2^ = 0.202. *C*, HPLC analysis of stability of tcEv3 16–56 dPG in human plasma at 37 °C.

The formation of a β-sheet by the spatially close N and C termini of tEv3 17–56 provides an opportunity to cyclize the peptide via the backbone. In previous studies it has been shown that β-hairpins capped with a β-turn d-Pro-Gly motif stabilizes short loops (32). To introduce this β-turn into tEv3 17–56, an additional N-terminal residue from the Evasin-3 sequence was added to the sequence to match the length of the N- and C-terminal regions. The resulting sequence containing the Evasin-3 region Asn^16^–Asn^56^ and the d-Pro-Gly sequence was synthesized by tert-butyloxycarbonyl solid-phase peptide synthesis and cyclized using intermolecular native chemical ligation. Subsequent oxidative folding resulted in truncated cyclic Evasin-3 16–56 with a d-Pro-Gly turn (designated as tcEv3 16–56 dPG) (Fig. 5*A*).

### Affinity of Evasin-3 variants

The affinity of Evasin-3 and its variants to CXCL8 was studied by SPR biosensor analysis using C-terminally biotinylated CXCL8 immobilized on a streptavidin-modified chip surface. Evasin-3 variants bound CXCL8 in a dose-dependent manner. Native Evasin-3 showed slower association and dissociation kinetics compared with truncated variants (Fig. 5*B* and Fig. S11). At higher Evasin-3 concentrations, no increase in response units signal and curvature were observed in the SPR sensorgrams, restricting further affinity analysis. Saturated binding analysis for truncated variants resulted in *K_d_* values of 27 and 13 nm for tEv3 17–56 and tcEv3 16–56 dPG, respectively.

### Plasma stability

The proteolytic stability of full-length Evasin-3 and its variants was tested in pooled human plasma from healthy volunteers (Fig. S12*A*). After 2 h of incubation at 37 °C in human plasma full-length Evasin-3 degraded nearly quantitatively, losing C-terminal Arg^66^ and Arg^65^ (ΔC_1_ and ΔC_2_, respectively). Moreover, after 18 h of incubation only ΔC_2_ could be detected in solution. In contrast to the full-length protein, the truncated Evasin-3 variants showed no detectable signs of degradation after 18 h of incubation in human plasma (Fig. 5*C* and Fig. S12*B*).

### PMN migration assay

**Figure 6.**
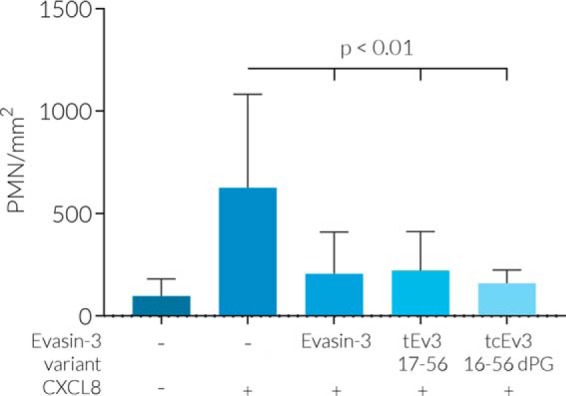
**Effect of Evasin-3 variants on CXCL8-induced PMN migration.** Shown is PMN migration as a result of the addition of 1 nm CXCL8 (*n* = 10) compared with the control without chemoattractant (*n* = 10) and compared with the effect of 10 nm Evasin-3 (*n* = 10), tEv3 17–56 (*n* = 9), or tcEv3 16–56 dPG (*n* = 5) on CXCL8-induced PMN migration.

To assess the anti-CXCL8 activity of Evasin-3 variants, polymorphonuclear neutrophil (PMN) migration assays were performed. The addition of 1 nm CXCL8 induced significant migration of PMNs (624 ± 458 PMN/mm^2^) compared with controls (98 ± 83 PMN/mm^2^) in the absence of chemoattractant (Fig. 6). Migration was inhibited by addition of 10 nm of Evasin-3 (206 ± 203 PMN/mm^2^). Significant effects were observed in the presence of tEv3 17–56 (223 ± 189 PMN/mm^2^) and tcEv3 16–56 dPG (160 ± 65 PMN/mm^2^).

### Identification of Evasin-3 homologues

A search against UniProtKB using the BLASTp server yielded 35 protein sequences with 69–39% sequence identity. Of these, fifteen were identified to come from *Ixodes* genus, twelve were from *Rhipicephalus*, seven were from *Amblyomma*, and one was from *Hyalomma*. In all cases, six cysteine positions were conserved, whereas hydrophobic residues in regions preceding C1 and following C5 and C6 residues (Fig. S13) appeared invariant.

## Discussion

Evasins are a family of tick salivary chemokine-binding proteins comprised of two subfamilies. Whereas Evasin-1 and Evasin-4 comprise eight cysteines, Evasin-3 contains six cysteines located in the compact core region. The 3D structure of the Evasin-3 core is largely determined by its three disulfide bonds. The Cys^26^–Cys^39^ bond protrudes through the ring comprised by the Cys^22^–Cys^37^ and Cys^33^–Cys^50^ forming an inhibitory cystine-knot (ICK) motif. Although the core sequence of Evasin-3 fits the consensus sequence for the ICK motif having five loops that are separated by conserved cysteines (33), no structural homology to known proteins could be found in DALI or in the Knottin3D database. The scaffold of Evasin-3 is predominantly composed by Thr^27^–Glu^32^ and Gly^40^–His^49^ loops. The Thr^27^–Glu^32^ loop is rigid, whereas the Gly^40^–His^49^ loop is relatively flexible. Because Evasin-3 binds closely related CXCL1 and CXCL8 chemokines and Gly^40^–His^49^ loop composes the great part of CXCL8–Evasin-3 complex interface, flexibility of this region could be necessary to fine-tune the spatial structure of the binding region during complex formation with different targets. The ICK motif is common for conotoxins (34), spider venoms, and cyclotides (35), but examples among tick proteins are rare and to our knowledge are limited to holocyclotoxins (36, 37). A database search identified 35 putative Evasin-3 homologues that share the inhibitory cystine knot motif. Identified sequences embody conservative regions of hydrophobic residues that compose the complex interface in the case of the CXCL8–Evasin-3 complex. That most likely indicates a similar chemokine-binding mode for these putative Evasins.

In contrast to Evasin-1, where both the N and C termini play a crucial role in chemokine binding (27, 38), neither the N nor C terminus of Evasin-3 participate in CXCL8 binding. Moreover, the N and C termini are predicted to be highly glycosylated (Fig. 1*C*), which is consistent with earlier observation that glycosylation of Evasin-3 does not play an essential role in chemokine binding (14). To study the effect of termini on binding, we synthesized two novel CXCL8-binding peptides: linear tEv3 17–56 and cyclic tcEv3 16–56 dPG, both retaining the ICK bundled core of the native protein. The ICK motif is known to contribute drastically to increase of protein and peptide stability under harsh extracellular conditions (39). The equally high proteolytic stability of the linear tEv3 17–56 and cyclic tcEv3 16–56 dPG in human plasma is in accordance with previous studies indicating that presence of a cystine knot is more vital for peptide stability than a cyclized backbone (40, 41). Although both tEv3 17–56 and cyclic tcEv3 16–56 dPG keep nanomolar affinity to CXCL8 (*K_d_* values of 27 and 13 nm, respectively), these *K_d_* values fall significantly above the 0.43 nm *K_d_* value previously measured for native Evasin-3 (14). Apparently, the presence of the long N and C termini has a direct effect on internal dynamics of Evasin-3 structure that causes slower association and dissociation rates of binding CXCL8 and thus lower *K_d_* values.

Final energy-minimized models of the complex between CXCL8 and tEv3 17–56 were calculated by HADDOCK using experimentally observed CXCL8–met-Evasin-3 contacts. It has been shown that Evasin-1 and Evasin-4 binding to chemokine N-terminal regions prevents their interactions with chemokine-receptor recognition site 2 (27, 28). Evasin-3 leaves both receptor-binding sites of CXCL8 unoccupied and available for possible alternative interactions (42–45). To test this hypothesis, PMN migration assays were performed. CXCL8 acted as a potent chemoattractant through interaction with its cognate CXCR1/2 receptors. However, the present data showed that CXCL8 bound to Evasin-3 or one of the truncated variants was unable to induce PMN migration and thus activate the CXCR2 receptor presented on the cell surface. This observation could be explained by structural changes in the N-loop of CXCL8, which residues are crucial for binding of the CXCR1/2 N-domain (46). Although Evasin-3 does not directly interact with the receptor-binding region of CXCL8 N-loop, significant perturbations were observed for Tyr^13^–Phe^17^ residues upon binding of Evasin-3 and the truncated variant tEv3 17–56. These perturbations were most likely caused by internal changes in CXCL8 packing upon formation of the complex. Indeed, Evasin-3 binding caused substantial repositioning of the α-helix and the N-loop compared with CXCL8 dimer and physiologically active obligatory CXCL8 monomers (42, 47).

According to the obtained model, the Cys^39^–Gln^44^ core region of Evasin-3 binds the CXCL8 β1-strand and the C-terminal helix, thereby partially blocking the CXCL8 dimer interface. Although Evasin-3 quickly binds to monomeric V27P/E29P CXCL8, SEC and NMR experiments showed that binding to native CXCL8 dimer is kinetically slow. That could indicate that the rate-limiting step in the CXCL8–Evasin-3 complex formation is the CXCL8 dimer dissociation, and the primary target of Evasin-3 is the CXCL8 monomer present in the bloodstream. This also means that influence of the chemokine oligomeric state should be taken into account during the assay design for research on chemokine-binding proteins that enables a more comprehensive view on chemokine/chemokine-binding protein relationships.

GAGs are believed to be storage for CXCL8 and orchestrate levels of free dimeric and monomeric CXCL8 (48). CXCL8 binding to GAGs is mediated through interactions with positively charged residues in the N-loop and the C-terminal α-helix of CXCL8 (29, 49). Flanking β-strands of Evasin-3 intercalate between the CXCL8 α-helix and the N-loop, preventing binding of CXCL8 to GAGs by disrupting the continuous stretch of positively charged residues of the CXCL8 GAG-binding site. Both NMR and SEC experiments showed that Evasin-3 can compete with GAG binding and slowly substitute GAGs from the CXCL8–GAGs complex. The rate-limiting step in this substitution apparently is release of monomeric CXCL8. As a result, Evasin-3 not only neutralizes active monomeric CXCL8 in the bloodstream but also depletes the storage of CXCL8 in the GAG-bound form.

In summary, 3D structures of Evasin-3 and its complex with chemokine CXCL8 have been studied by solution NMR spectroscopy. Evasin-3 represents a rare example of a tick protein with an inhibitory cysteine knot motif. Tight binding of Evasin-3 to the CXCL8 monomer blocks interactions with CXCR2 and disrupts the GAG-binding interface, leading to elimination of both chemotactic and haptotactic gradients. Two novel CXCL8-binding peptides were derived from the Evasin-3 sequence: linear tEv3 17–56 and cyclic tcEv3 16–56 dPG. These short variants are easily accessible by chemical synthesis combining high affinity to CXCL8 with great proteolytic stability. Importantly, synthetic Evasin-3 variants can be used as a scaffold for design and fine-tuning of new selective chemokine-binding agents.

## Experimental procedures

### Expression of recombinant proteins

The pET23a vector containing human CXCL8 (UniProt: p10145, 6–77) and monomeric variant V27P/E29P CXCL8 and pET30a containing Evasin-3 (UniProt: p0c8e8, 1–66) genes were purchased from GenScript. Both proteins were expressed in BL21 (DE3) Star (Novagen). The cells were grown in 1 liter of standard LB medium at 37 °C in the presence of the required antibiotic. Once the *A*_600_ value reached 0.6–0.8, expression of protein was induced with 0.1 or 1 mm of isopropyl β-d-1-thiogalactopyranoside (Sigma–Aldrich) in the case of pET23a and pET30a vector, respectively. The cells were harvested 3 h after induction by centrifugation at 4,000 rpm for 20 min at 4 °C.

To obtain [^15^N,^13^C]-labeled proteins, the cells were grown in LB medium as described above. After *A*_600_ reached 0.6–0.8, the cells were harvested by centrifugation at 4,000 rpm at 4 °C and transferred to M9 medium. Prior to induction with isopropyl β-d-1-thiogalactopyranoside, the cells were incubated in medium for 1 h to deplete internal carbon and nitrogen sources. At induction, the medium was supplemented with 1 g/liter of [^13^C]glucose and ^15^NH_4_Cl, incubated for 3 h, and harvested by centrifugation.

The cells were resuspended in 50 mm Tris, pH 8, and lysed with 1× Bugbuster (Novagen) and 0.1 unit/ml Benzonaze® nuclease (Sigma–Aldrich). For CXCL8, the cells were lysed, and the supernatant was discarded by centrifugation at 10,000 rpm for 20 min at 4 °C. Insoluble fraction was washed twice with 50 mm Tris, pH 8, and once with 50 mm Tris, pH 8, 0.5% Tween 20. Insoluble pellets were dissolved in 0.1 mm Tris, pH 8, 200 mm DTT, 6 m guanidine HCl, and dialyzed overnight against 0.5% acetic acid using a 3.5-kDa spectra/Por RC membrane (Repligen). In the case of Evasin-3, cell debris was removed by centrifugation at 10,000 rpm for 20 min at 4 °C, and the soluble fraction was dialyzed as described above. The soluble fractions after dialysis were centrifuged at 10,000 rpm for 20 min at 4 °C and then lyophilized.

Lyophilized material was dissolved in 6 m guanidine HCl, 0.1 mm Tris, pH 8, at 20 mg/ml and then added dropwise to 1 m guanidine HCl, 0.1 mm Tris, pH 8, 10 mm cysteine, 1 mm cystine, at 4 °C to a final concentration of 1 mg/ml. Folded proteins were purified by HPLC using 22-mm × 250-mm Vydac C18 columns, analyzed by LC-MS, and lyophilized.

### Peptide synthesis

tEv3 17–56 and tcEv3 16–56 dPG were synthesized by tert-butyloxycarbonyl solid-phase peptide synthesis and folded as described previously (31). For one-pot intramolecular cyclization and folding, crude tcEv3 16–56 dPG was dissolved at a concentration of 0.2 mg/ml in 50 mm ammonium bicarbonate buffer, pH 8. After completion of the reaction, the folded cyclic peptide was purified by HPLC and lyophilized.

### Surface plasmon resonance

Affinity assays of Evasin-3 variants were performed using BIAcore T200 surface plasmon resonance system (GE Healthcare). All assays were carried out in PBS buffer, pH 7.4, supplemented with 0.05% Tween 20 as running buffer. 70 response units of chemically synthetized CXCL8-Biotin was immobilized on a SAHC 200M sensorchip (Xantec) according to the manufacturer's protocol. Concentration series of Evasin-3 variants, ranging from 1 μm to 2 nm, were prepared by 2-fold dilution of 50 μm stock solutions. Association and dissociation kinetics were monitored at flow rate 30 μl/min for 250 and 300 s, respectively. The data were analyzed using BIAcore T200 software and BIAEvaluation software (GE Healthcare) using a steady-state affinity equation with a linear component.

### NMR

NMR samples were prepared from freeze-dried proteins as 0.2 mm solutions in 25 mm deuterated NaAc-d^3^ buffer, pH 4.5, containing 0.1 mm EDTA, 0.2 mm sodium azide, and 5% (v/v) D_2_O for deuterium lock. To remove TFA salts presented in freeze-dried samples of proteins, initial buffer exchange was carried out by ultracentrifugation (in four or five steps) using prewashed Amicon Ultra-3000 3-kDa (Millipore) ultracentrifugation filters. Final NMR solutions were prepared in Wildman 3-mm NMR tubes (160 μl volume), containing traces of 4,4-dimethyl-4-silapentane-1-sulfonic acid for internal chemical shift calibration (0 ppm ^1^H).

All NMR spectra were recorded using Bruker Avance III HD 700 MHz spectrometer, equipped with a cryogenically cooled TCI probe. Sample temperature was set to 25, 37, or 44 °C, with the accurate probe temperature calibrated using a thermocouple inside an NMR tube that was inserted into the probe. Backbone resonance assignment of met-Evasin-3, tEv3 17–56, and CXCL8 was derived from a combination of ^15^N-^1^H HSQC, HNCO, HNCACO, HNCACB, and CBCA(CO)NH spectra. Aliphatic side chain resonances were assigned using ^13^C-^1^H HSQC and hCCH DIPSI spectra. Distance constraints were extracted from ^15^N and ^13^C NOESY spectra.

To study the Evasin-3–CXCL8 1:1 complex, two different samples were prepared with either [^15^N,^13^C]met-Evasin-3 or [^15^N,^13^C]CXCL8 as labeled component at the final concentration of 200 μm. Titrated complexes contain a slight excess of unlabeled CXCL8 or met-Evasin-3, respectively. After completion of complex formation, a set of triple resonance spectra, as described above, were recorded for both met-Evasin-3 and CXCL8 in the bound form. Intramolecular contacts of CXCL8 and met-Evasin-3 were obtained from filtered ^15^N and ^13^C NOESY experiments.

For GAG-binding experiments low-molecular-mass heparin dalteparin (commercial name Fragmin®, Pfizer) and fondaparinux (commercial name Arixtra®, GSK) were purchased from a pharmacy as solutions for injections. 25 μm of [^15^N,^13^C]CXCL8 was titrated by increasing concentration of aliquots from a stock solution of 5 mg/ml fondaparinux or Fragmin to the final GAG concentration of 0.2 mm at 25 °C, pH 7.1. In the next step, 30 μm of met-Evasin-3 was added to the solution to monitor the displacement of bound fondaparinux and follow Evasin-3–CXCL8 complex formation by a series of 1D proton and ^15^N-^1^H HSQC spectra.

Spectra processing was performed by Bruker Topspin 3.2 and Sparky 3.114 software. The secondary structure was predicted by CSI 3.0 webserver (50). Structure calculations were performed by dynamics simulated annealing method using Xplor-NIH software (51, 52) and refined via the NMRe web server (53). Structure refinement of the complex was performed using HADDOCK 2.2 server (54).

### Size-exclusion chromatography

SEC experiments were carried out on a Varian ProStar 215 solvent delivery system, coupled to a Varian ProStar 320 UV/VIS detector set at a wavelength of 280 nm. Separation was performed using a BioSep 5-μm SEC-s2000 145 Å, 300 × 4.6-mm LC column (Phenomenex, Torrance, CA). 50 mm sodium phosphate buffer, pH 6.65, containing 150 mm NaCl was used as eluent buffer. Flow rate was set at 0.5 ml/min. Proteins were injected at 0.1 mg/ml concentration in the presence or absence of 1 mg/ml low-molecular-mass heparin (commercial name Fragmin®, Pfizer). Molecular masses of proteins were calculated using the calibration curve provided by the manufacturer.

### Plasma stability

100 μg of Evasin-3 variants were dissolved in 100 μl of normal plasma taken from a healthy volunteer and incubated at 37 °C. At a required time point reaction was stopped by addition of 15 μl of 20% TCA to a 15-μl aliquot of a sample and analyzed by LC-MS.

### Bioinformatics

Glycosylation sites were predicted using NetNGlyc 1.0 and NetOGlyc 4.0 servers. The threshold for both positive *N*- and *O-*glycosylation sites was set at 0.5. For homology search, Evasin-3 sequence (UniProt ID p0c8e8) was used as a query for BLASTp algorithm against UniProtKB database (55). Multiple sequence alignment was performed using Clustal Omega algorithm (56) with default settings, and the results were visualized by JalView 2.10 software.

### PMN isolation and migration

Human neutrophils were isolated from the blood of healthy donors as described previously (57). Isolated neutrophils were resuspended at (10^6^ cells/ml) in RPMI 1640 medium supplemented with 1% fetal calf serum. A 12-well chemotaxis chamber with a 5-μm polycarbonate membrane (Neuroprobe, Gaithersburg, MD) was used to assess the migration of neutrophils toward the chemoattractant CXCL8 (1 nm) in presence or absence of Evasin-3 variants (10 nm). The chemoattractant was added in the lower wells, and the neutrophils (1 × 10^5^ cells) were seeded in the upper wells. After incubation of 90 min at 37 °C, the nonmigrated cells were carefully removed, and the membrane was stained with Diff-Quick stain (Eberhard Lehmann GmbH, Berlin, Germany). Migrated cells were visualized by light microscopy, counted manually in three fields of view, and expressed as cells/mm^2^. The data were analyzed by GraphPad Prism 8.0 software using one-way analysis of variance statistical test.

## Author contributions

S. S. D. and I. D. conceptualization; S. S. D. formal analysis; S. S. D., J. H. I., A.C.A. H., and A.O.-G. investigation; S. S. D. visualization; S. S. D. and I. D. writing-original draft; J. H. I. data curation; J. H. I., T. M. H., and I. D. writing-review and editing; R. R. K., O. S., T. M. H., and I. D. supervision.

## Supplementary Material

Supporting Information

## Footnotes

2 The abbreviations used are:

GPCR: G protein-coupled receptor
CXCL8: chemokine (C*X*C motif) ligand 8
CXCL1: chemokine (C*X*C motif) ligand 1
CXCR2: C*X*C chemokine receptor 2
GAG: glycosaminoglycan
PMN: polymorphonuclear neutrophil
SPR: surface plasmon resonance
HSQC: heteronuclear single quantum coherence
ICK: inhibitory cystine-knot
SEC: size-exclusion chromatography
PDB: Protein Data Bank.
