# Supplementary material for: Tick saliva protein Evasin-3 modulates chemotaxis by disrupting CXCL8 interactions with glycosaminoglycans and CXCR2
Source: J Biol Chem. 2019 Jun 24;294(33):12370–9. doi: 10.1074/jbc.RA119.008902 (PMC6699855; doi:10.1074/jbc.RA119.008902)
Supplement: Supporting Information [file supp_294_33_12370__index.html]

Tick saliva protein Evasin-3 modulates chemotaxis by disrupting CXCL8 interactions with glycosaminoglycans and CXCR2 — Evasin-3-based peptides modulate chemotaxis by CXCL8 binding — Tick saliva protein Evasin-3 modulates chemotaxis by disrupting CXCL8 interactions with glycosaminoglycans and CXCR2 — Evasin-3-based peptides modulate chemotaxis by CXCL8 binding — Supporting Information 

# Tick saliva protein Evasin-3 modulates chemotaxis by disrupting CXCL8 interactions with glycosaminoglycans and CXCR2

## Supporting Information

- Supporting Information (to be published online) - NMR spectra, SEC, SPR analysis, plasma stability
