# Supplementary material for: Tick saliva protein Evasin-3 modulates chemotaxis by disrupting CXCL8 interactions with glycosaminoglycans and CXCR2
Source: J Biol Chem. 2019 Jun 24;294(33):12370–9. doi: 10.1074/jbc.RA119.008902 (PMC6699855; doi:10.1074/jbc.RA119.008902)
Supplement: Supporting Information [file supp_RA119.008902_144935_2_supp_346163_psxhm1.pdf]

Supporting information

Tick saliva protein Evasin-3 modulates chemotaxis by disrupting CXCL8 interactions with glycosaminoglycans and CXCR2.

Stepan S. Denisov[1], Johannes H. Ippel[1], Alexandra C.A. Heinzmann[1], Rory R. Koenen[1], Almudena Ortega-Gomez[2], Oliver Soehnlein[2,3,4,5], Tilman M. Hackeng[1], Ingrid Dijkgraaf[1]*

Figure S1. Overview of recombinantly expressed Evasin-3 folding and purification. S2

Figure S2. ^{15}N-^{1}H HSQC spectra of 200 µM [^{15}N, ^{13}C] met-Evasin-3 in free form and in the CXCL8/[^{15}N, ^{13}C] met-Evasin-3 complex at 44°C, pH 4.5. ....S3

Figure S3. ^{15}N-^{1}H HSQC spectra of 200 µM [^{15}N, ^{13}C] CXCL8 ....S4

Figure S4. ^{1}H strip plots from 3D ^{15}N NOESY spectra of 200 µM [^{15}N, ^{13}C] CXCL8 ....S5

Figure S5. Ribbon representation of the ensemble of 10 lowest energy structures of [^{15}N, ^{13}C] CXCL8 in the [^{15}N, ^{13}C] CXCL8/met-Evasin-3 complex S6

Figure S6. Contacts between met-Evasin-3 and CXCL8 ....S7

Figure S7. The section of ^{15}N-^{1}H HSQC spectra of 25 µM [^{15}N, ^{13}C] CXCL8 showing binding-induced chemical shift perturbations at different concentrations of Fondaparinux S8

Figure S8. The section of ^{15}N-^{1}H HSQC spectra of 25 µM [^{15}N, ^{13}C] CXCL8 ....S9

Figure S9. Chromatographic elution profiles S10

Figure S10. The chemical shift perturbation plot of 200 µM [^{15}N, ^{13}C] CXCL8 amide peaks upon binding with met-Evasin-3 and tEv3 17-56 ....S11

Figure S11. SPR biosensor analysis of met-Evasin-3 and tEv3 17-56..S12

Figure S12. HPLC analysis of stability of Evasin-3 and tEv3 17-56 in human plasma S13

Figure S13. Multiple sequence alignment of identified Evasin-3 homologues. S14

Table S1. HADDOCK report for the top cluster of the docked CXCL8/tEv3 17-56 model........................S15

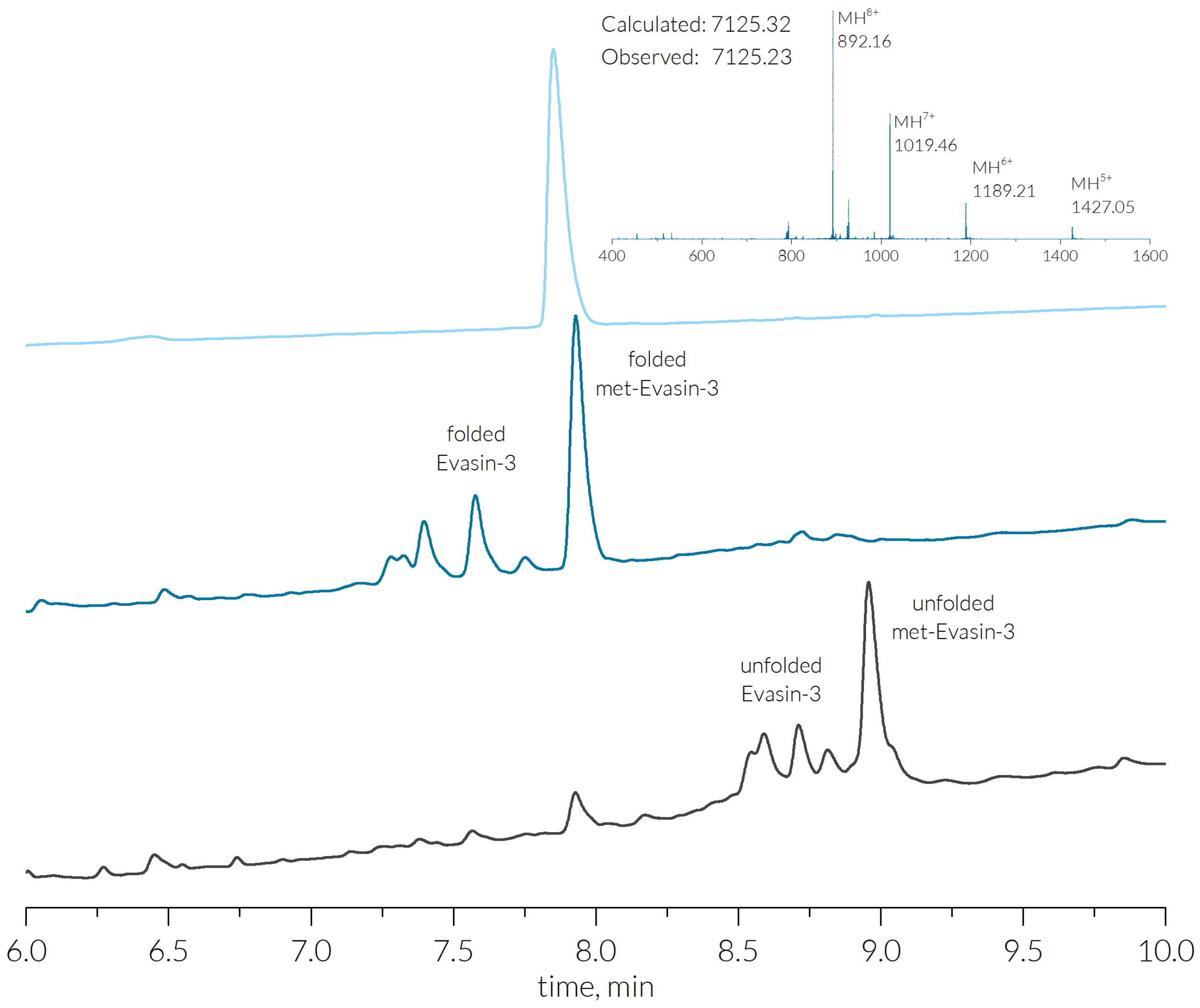

Figure S1. Overview of recombinantly expressed Evasin-3 folding and purification. The chromatograms of crude cell lysate before (black) and after (dark blue) folding. Results of the LC-MS analysis of purified met-Evasin-3 is shown in light blue.

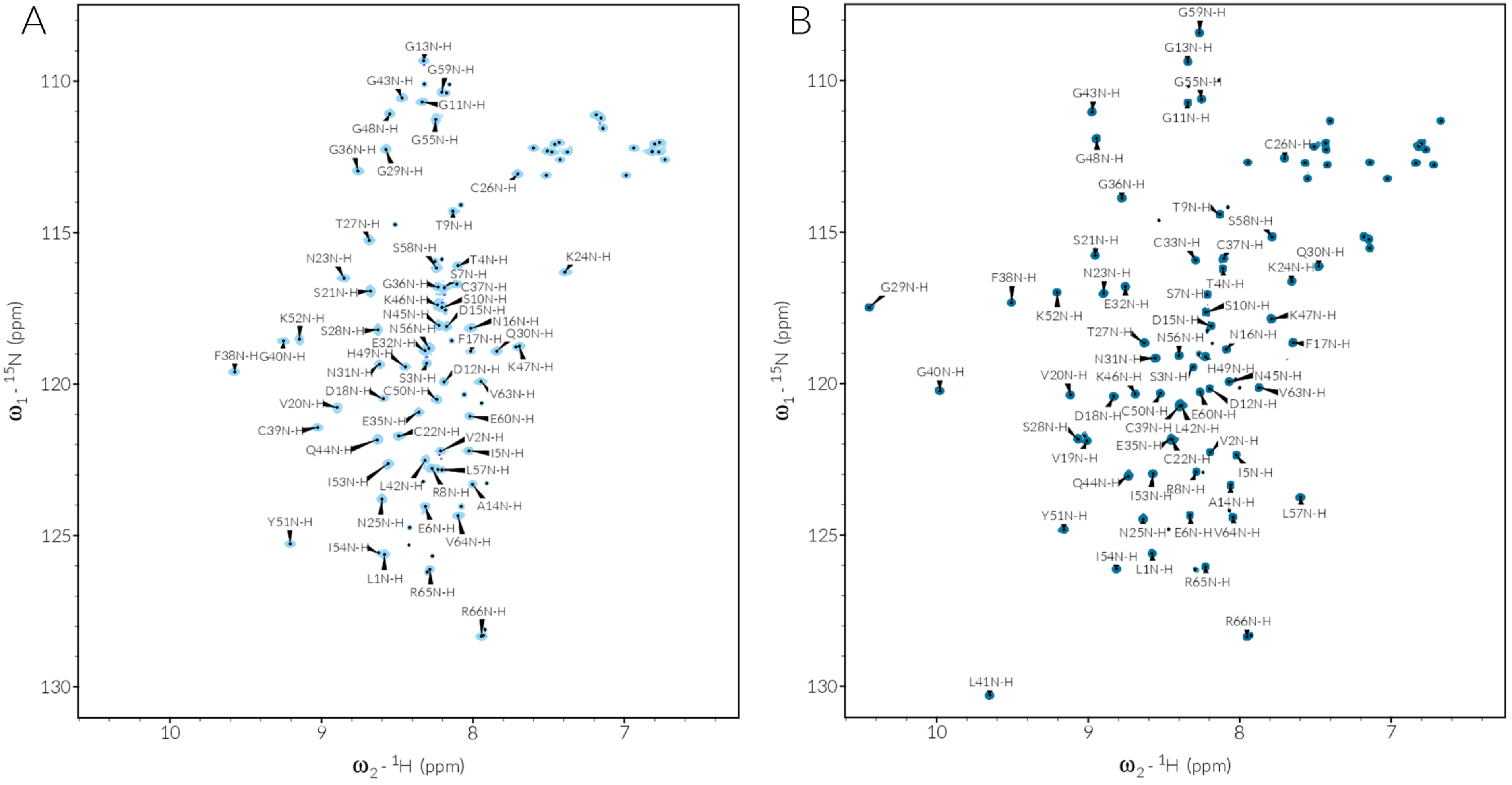

Figure S2. ^{15}N-^{1}H HSQC spectra of 200 μM [^{15}N, ^{13}C] met-Evasin-3 in free form (A) and in the CXCL8/[^{15}N, ^{13}C] met-Evasin-3 complex (B) at 44°C, pH 4.5. Side chain amide peaks are hidden for better visibility.

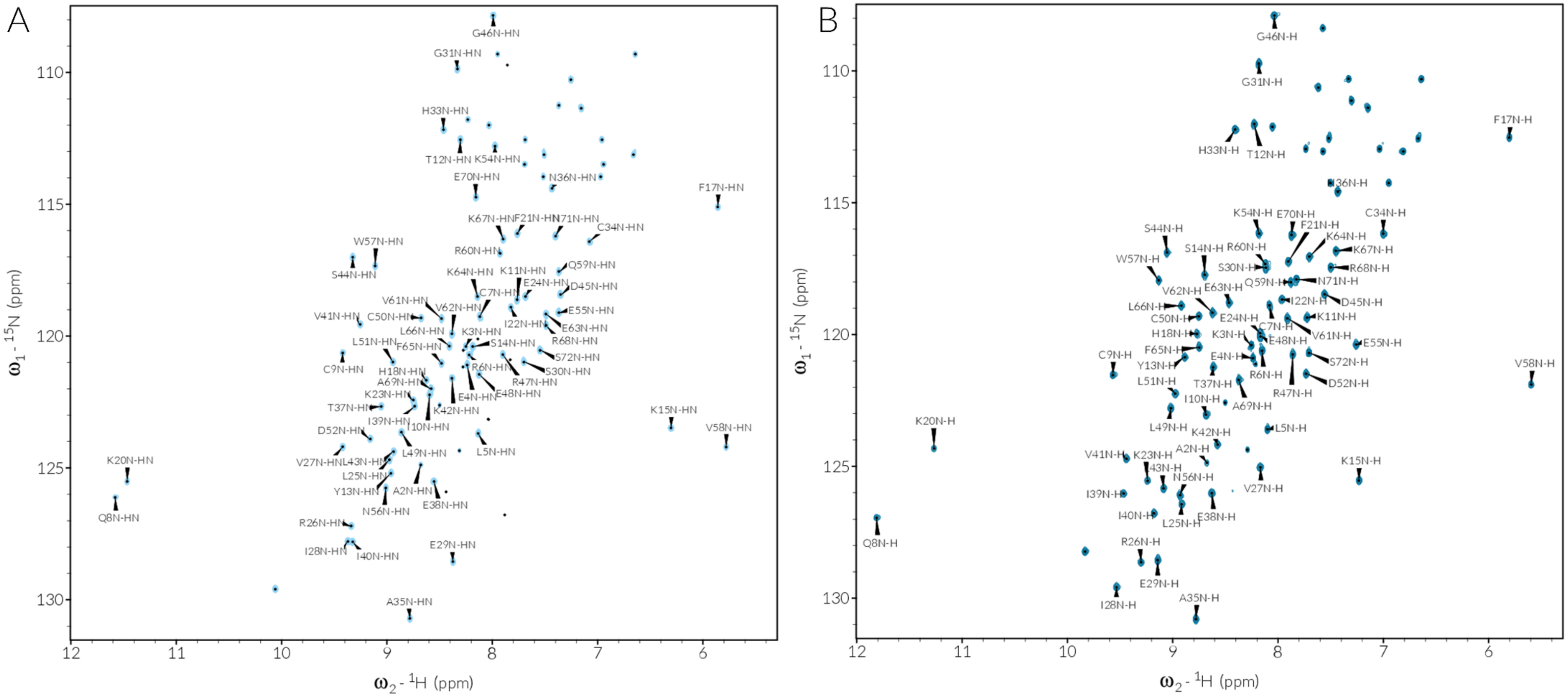

Figure S3. ^{15}N-^{1}H HSQC spectra of 200 μM [^{15}N, ^{13}C] CXCL8 in free form (A) and in the [^{15}N, ^{13}C] CXCL8/met-Evasin-3 complex (B) at 37°C, pH 4.5. Side chain amide peaks are hidden for better visibility.

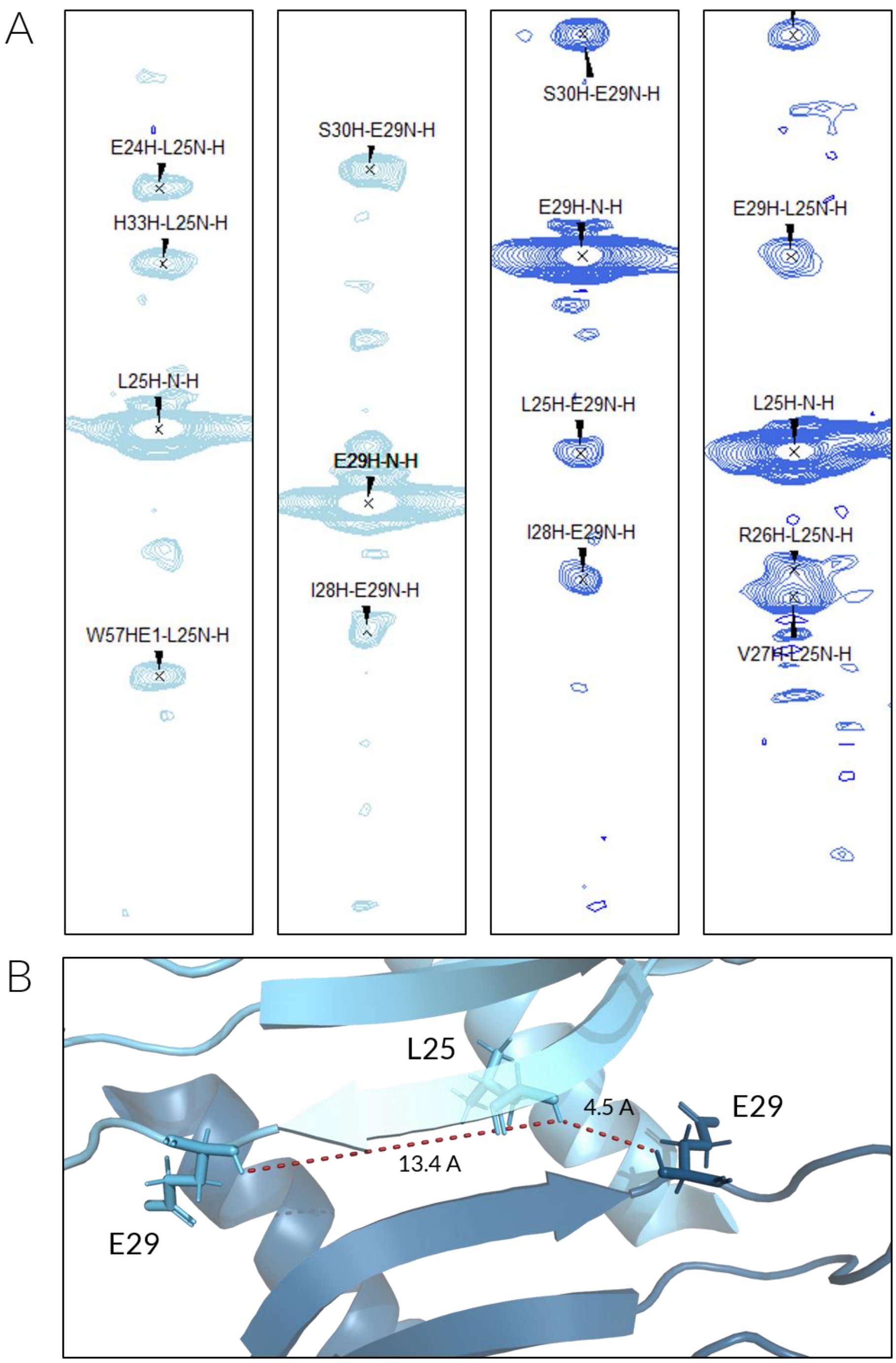

Figure S4. A. ^{1}H strip plots from 3D ^{15}N NOESY spectra of 200 μM [^{15}N, ^{13}C] CXCL8 in free form (light blue) and in the [^{15}N, ^{13}C] CXCL8/met-Evasin-3 complex (dark blue) at 37°C, pH 4.5. **B.** Intra- and intermolecular L25-E29 distances in CXCL8 dimer according the crystal structure (PDB ID 1IL8).

A

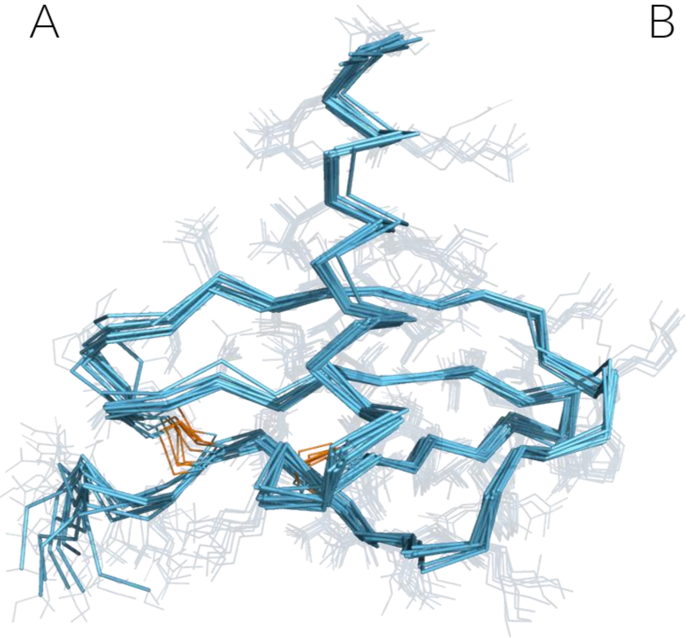

B

Experimental constraints	
Long	189
Medium *[1 < (i-j) <5]*	150
Sequential *[(i-j)=1]*	345
Intraresidue *[i=j]*	343
Total	1027
Dihedral angle constraints	114
Number of restrains per residue	15.8
Number of long-range restrains per residue	2.6
**Average atomic RMSD to the mean structure (Å)**	
Backbone	0.8
Heavy atoms	1.2
**Global quality scores (mean/Z score)**	
Verify3D	0.30/-2.57
Prosall	0.50/-0.62
PROCHECK (φ–ψ)	-0.11/-0.12
PROCHECK (all)	-0.17/-1.01
MolProbity clash score	0.67/1.41
**Ramachandran statistics (% of all residues)**	
Most favored	90.2
Additionally allowed	8.9
Generously allowed	0.3
Disallowed regions	0.6

C

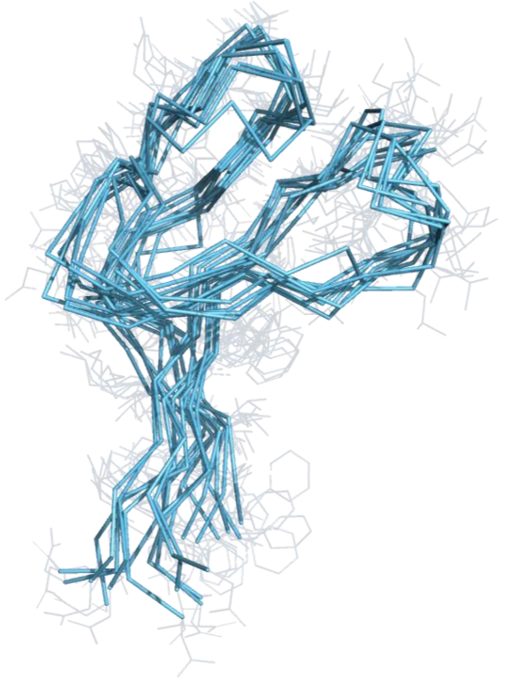

D

Experimental constraints		
Region	1-66	17-56
Long	84	71
Medium *[1 < (i-j) <5]*	35	28
Sequential *[(i-j)=1]*	173	138
Intraresidue *[i=j]*	185	141
Total	477	376
Dihedral angle constraints	64	60
Number of restrains per residue	8.2	10.9
Number of long-range restrains per residue	1.3	1.8
**Average atomic RMSD to the mean structure (Å)**		
Backbone	3.9	2.0
Heavy atoms	4.6	2.5
**Global quality scores for 17-56 aa (mean/Z score)**		
Verify3D	0.29/-2.73	
Prosall	0.28/-1.53	
PROCHECK (φ–ψ)	-1.65/-6.18	
PROCHECK (all)	-1.72/-10.17	
MolProbity clash score	109.58/-17.28	
**Ramachandran statistics (% of all residues)**		
Most favored	53.9	
Additionally allowed	40.0	
Generously allowed	3.3	
Disallowed regions	2.7	

Figure S5. A. Ribbon representation of the ensemble of 10 lowest energy structures of [^{15}N, ^{13}C] CXCL8 in the [^{15}N, ^{13}C] CXCL8/met-Evasin-3 complex; disulfides are colored in yellow. **B.** NMR and refinement statistics for [^{15}N, ^{13}C] CXCL8 structure in the [^{15}N, ^{13}C] CXCL8/met-Evasin-3 complex. **C.** Ribbon representation of the ensemble of 10 lowest energy structures of the F17-N56 region of [^{15}N, ^{13}C] met-Evasin-3 in the CXCL8/[^{15}N, ^{13}C] met-Evasin-3 complex. **D.** NMR and refinement statistics for met-Evasin-3 structure in the CXCL8/[^{15}N, ^{13}C] met-Evasin-3 complex.

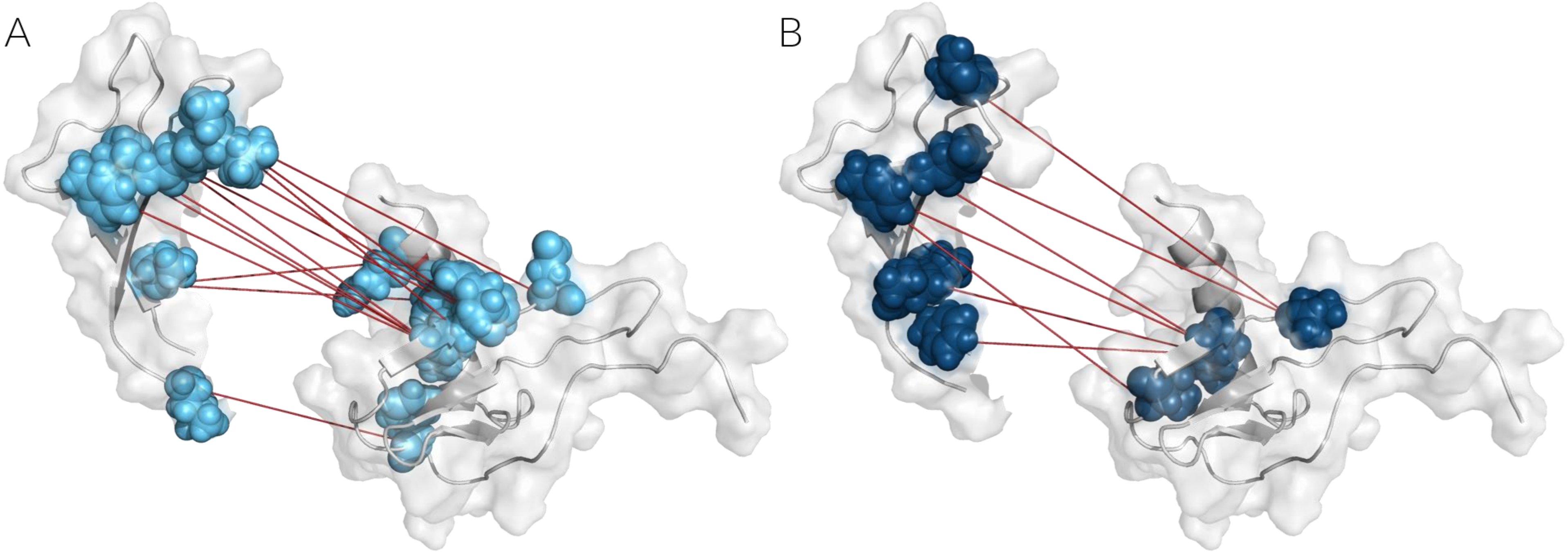

Figure S6. Contacts between met-Evasin-3 and CXCL8 derived from ^{15}N (A) and ^{13}C (B) filtered NOESY spectra of 200 µM [^{15}N, ^{13}C] CXCL8/met-Evasin-3 complex at 37°C, pH 4.5. N- and C-termini of met-Evasin-3 are hidden for better visibility.

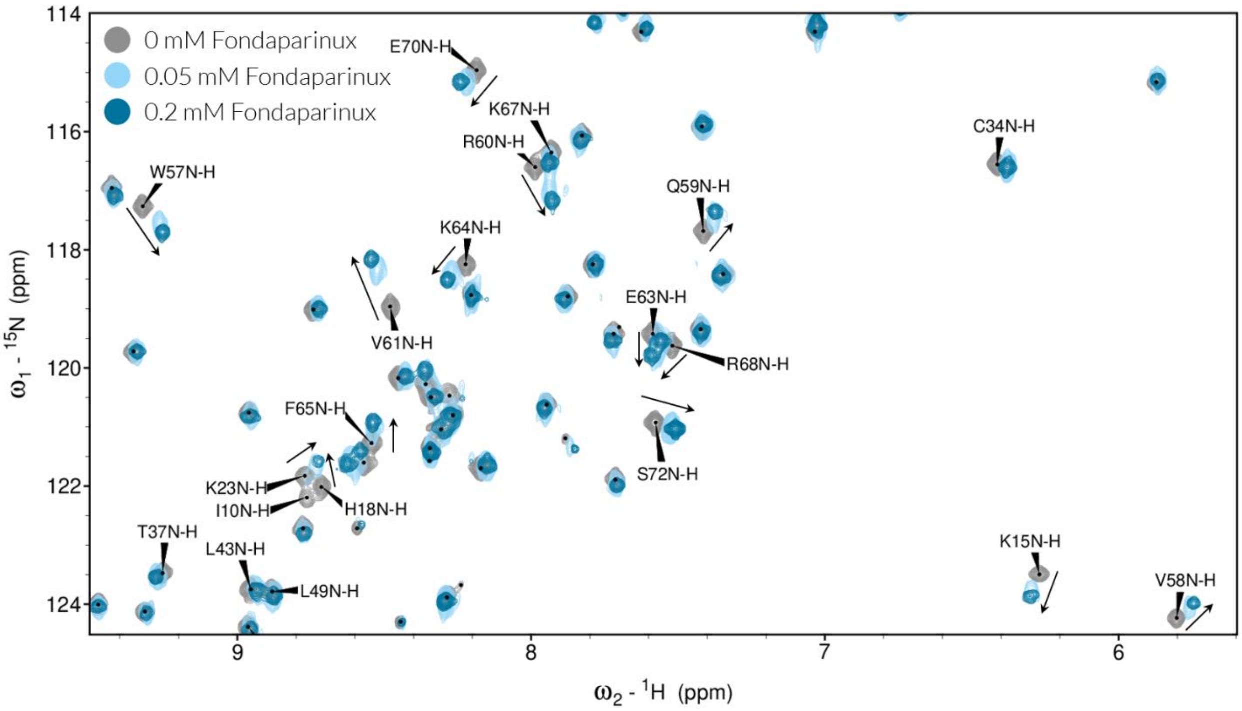

Figure S7. The section of ^{15}N-^{1}H HSQC spectra of 25 μM [^{15}N, ^{13}C] CXCL8 showing binding-induced chemical shift perturbations at different concentrations of Fondaparinux at 25°C, pH 7.1. Arrows indicate the direction of peak movement.

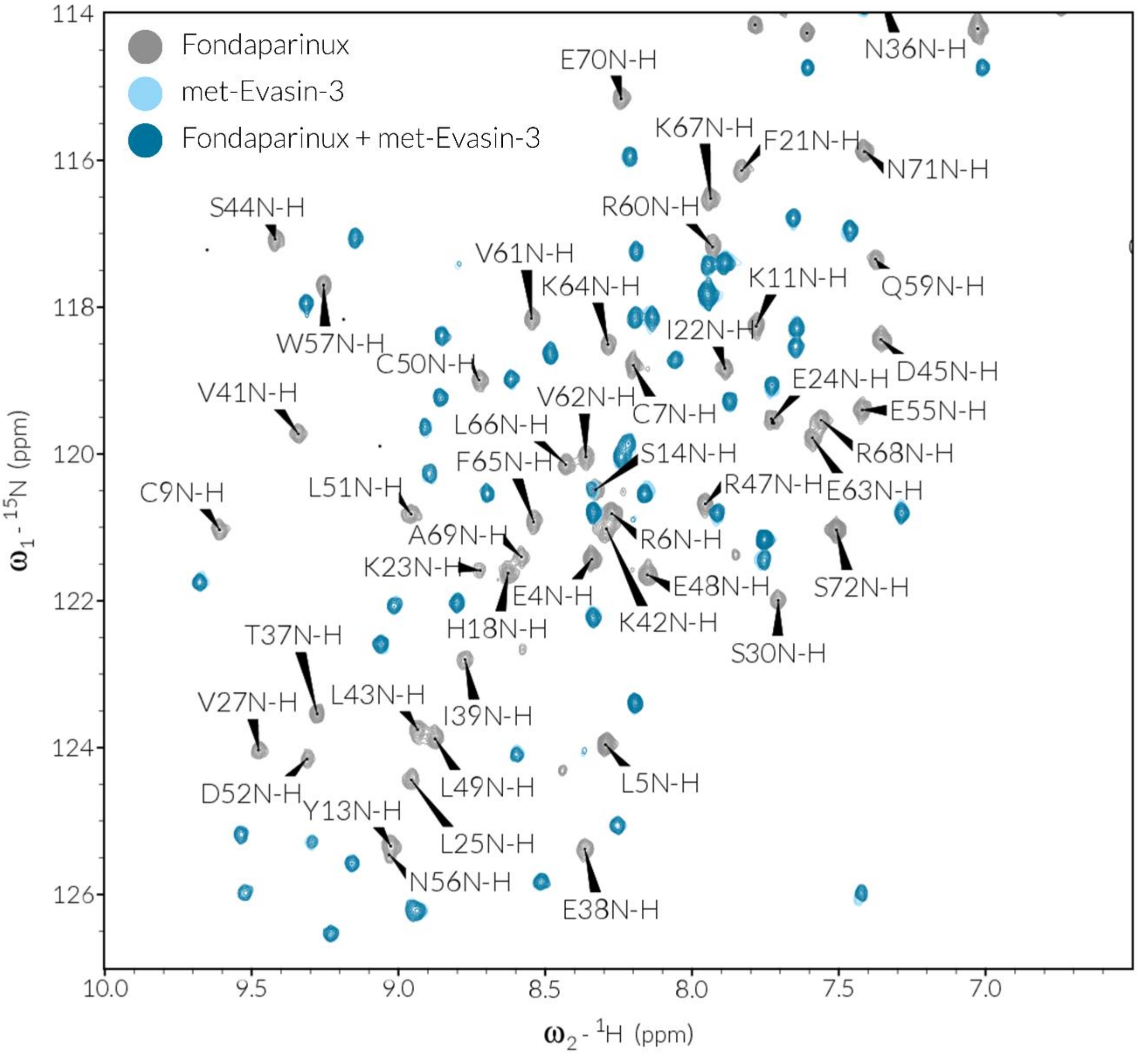

Figure S8. The section of ^{15}N-^{1}H HSQC spectra of 25 μM [^{15}N, ^{13}C] CXCL8 in the presence of 200 μM of Fondaparinux complex (grey), 30 μM met-Evasin-3 complex (light blue), and in the presence of 200 μM of Fondaparinux (Arixtra®, GSK) after addition of 30 μM met-Evasin-3 (dark blue) at 25°C, pH 7.1. Only assignment of [^{15}N, ^{13}C] CXCL8/Fondaparinux complex are shown for better visibility.

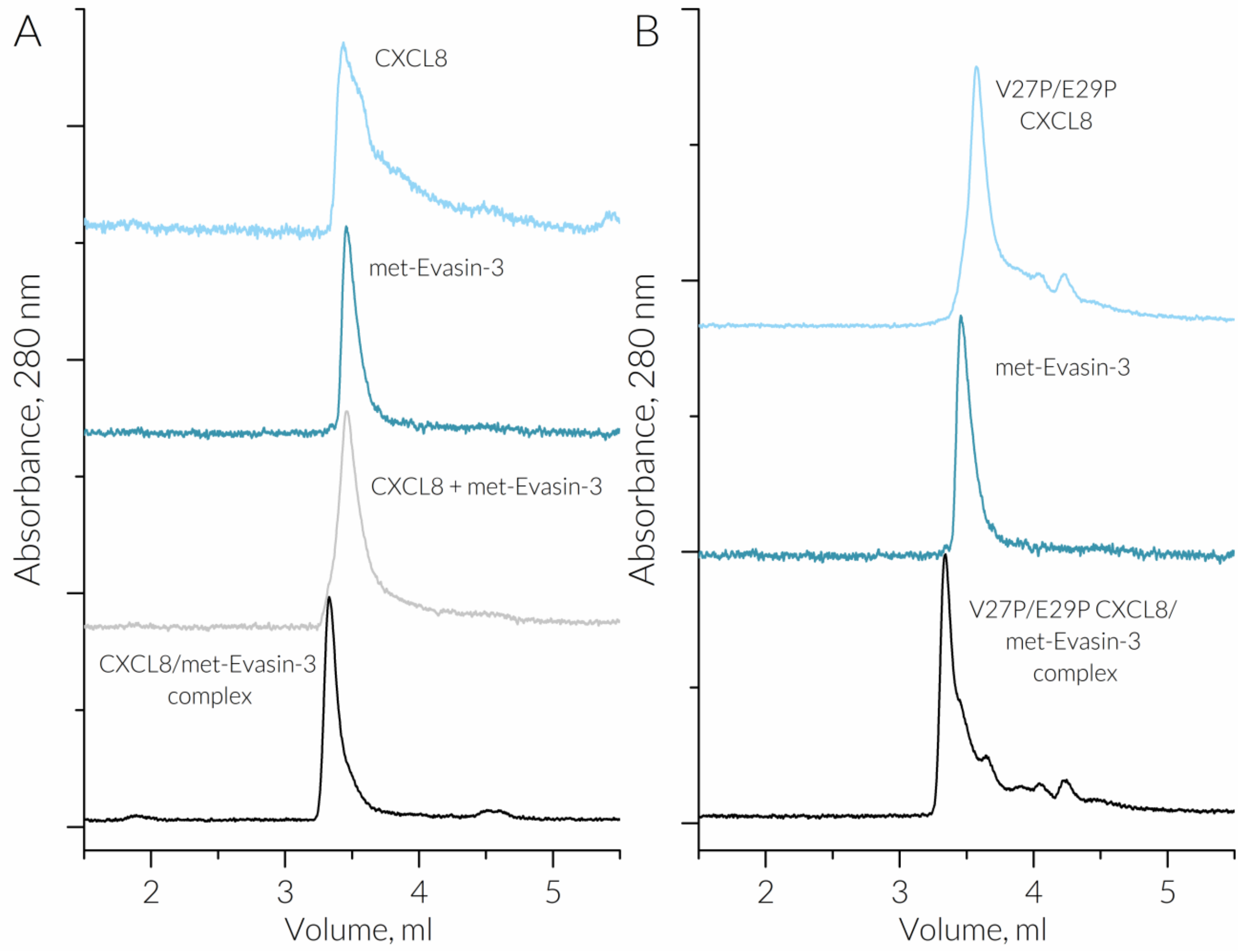

Figure S9. A. Chromatographic elution profiles of 0.1 mg/ml CXCL8 (light blue), 0.1 mg/ml of met-Evasin-3 (dark blue) and their mixture at t=0 (grey) and after 1-hour incubation at 37°C (black). **B**. Chromatographic elution profiles of 0.1 mg/ml V27P/E29P CXCL8 (light blue), 0.1 mg/ml of met-Evasin-3 (dark blue) and their mixture at t=0 (grey).

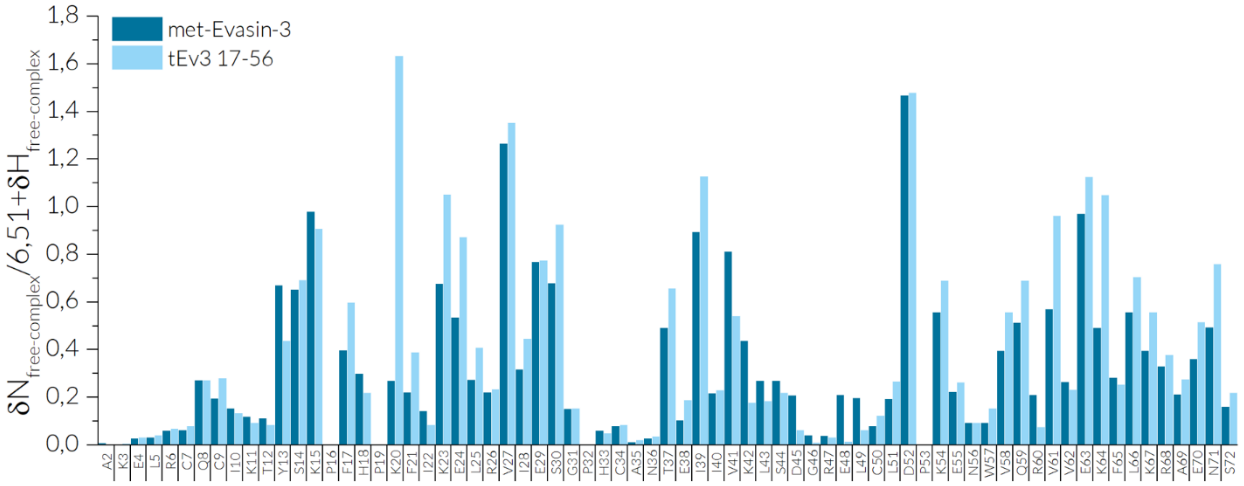

Figure S10. The chemical shift perturbation plot of 200 μM [^{15}N, ^{13}C] CXCL8 amide peaks upon binding with met-Evasin-3 (dark blue) and tEv3 17-56 (light blue) at 37°C, pH 4.5.

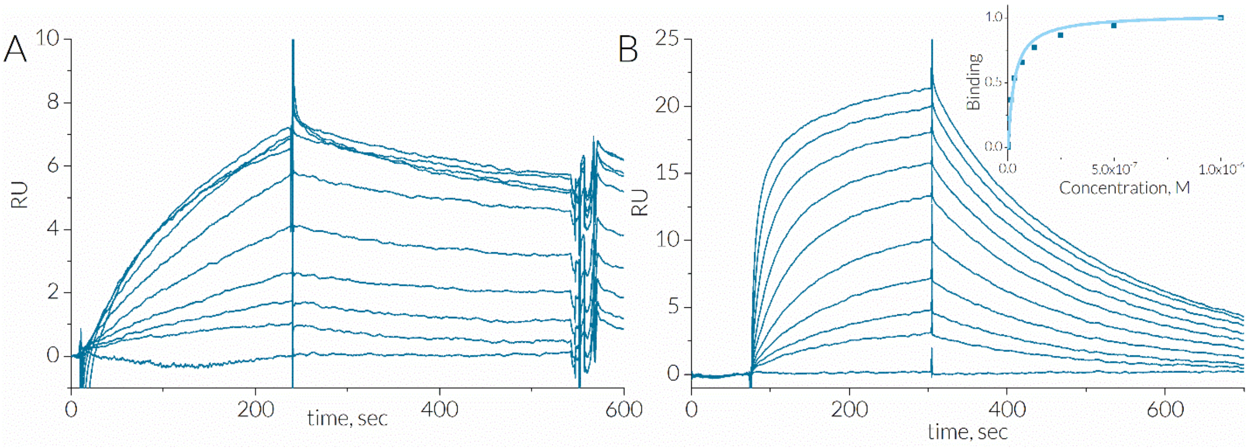

Figure S11. SPR biosensor analysis of met-Evasin-3 (A) and tEv3 17-56 upon binding to immobilized human CXCL8. The binding curve for tEv3 17-56 is plotted using maximal response signal for each injection. Apparent Kd value is calculated by fitting the data to a steady-state affinity model using a linear component. C. HPLC analysis of stability of tcEv3 16-56 dPG in human plasma at 37°C. Fitted Kd is 27 nM, R_{max} = 20.5, Chi^2 = 0.285.

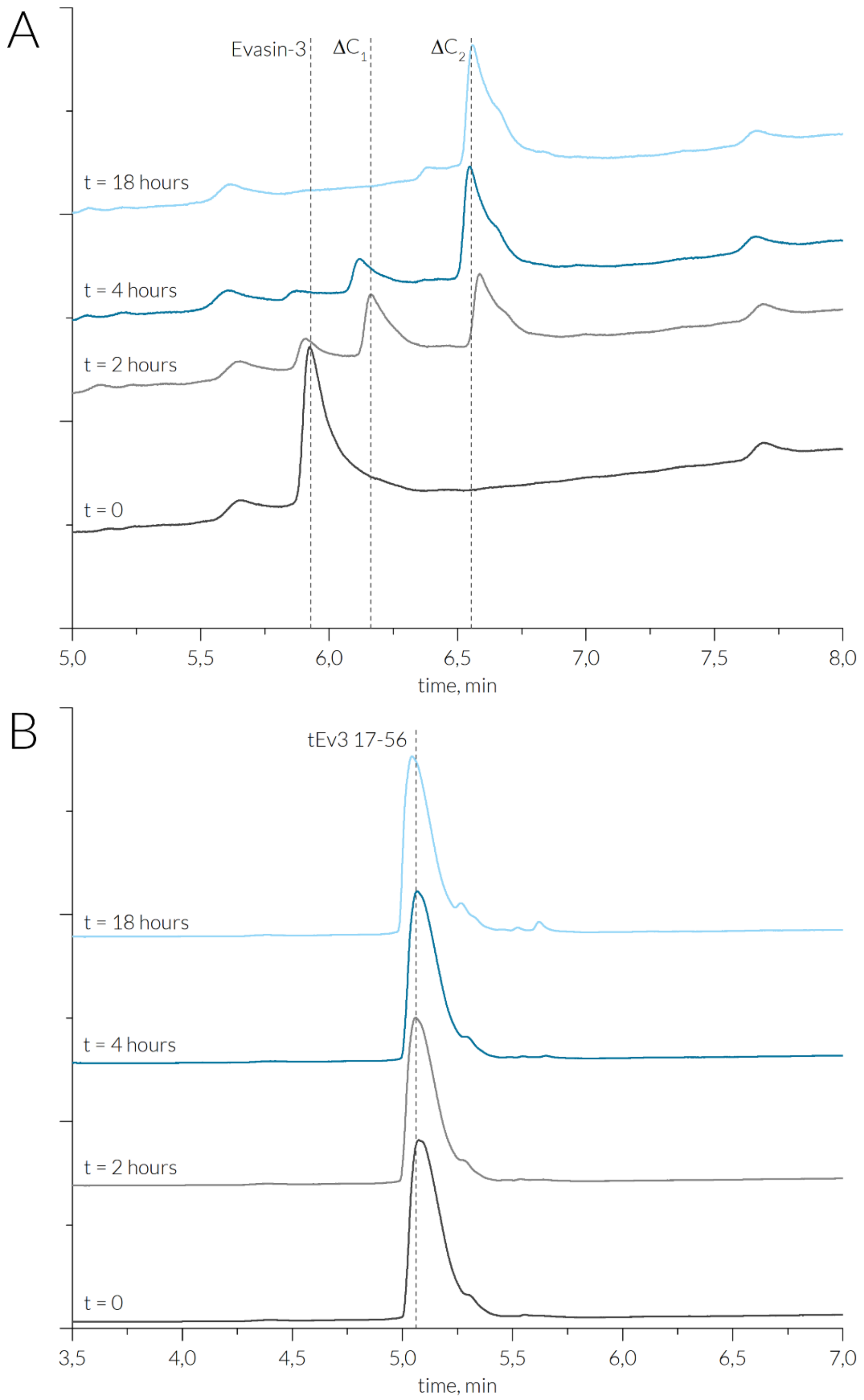

Figure S12. HPLC analysis of stability of Evasin-3 (A) and tEv3 17-56 (B) in human plasma at 37°C. Mass difference for ΔC_1 and ΔC_2 comparing with Evasin-3 was 156.1 and 312.3 Da corresponding to removal of one and two C-terminal Arg residues, respectively.

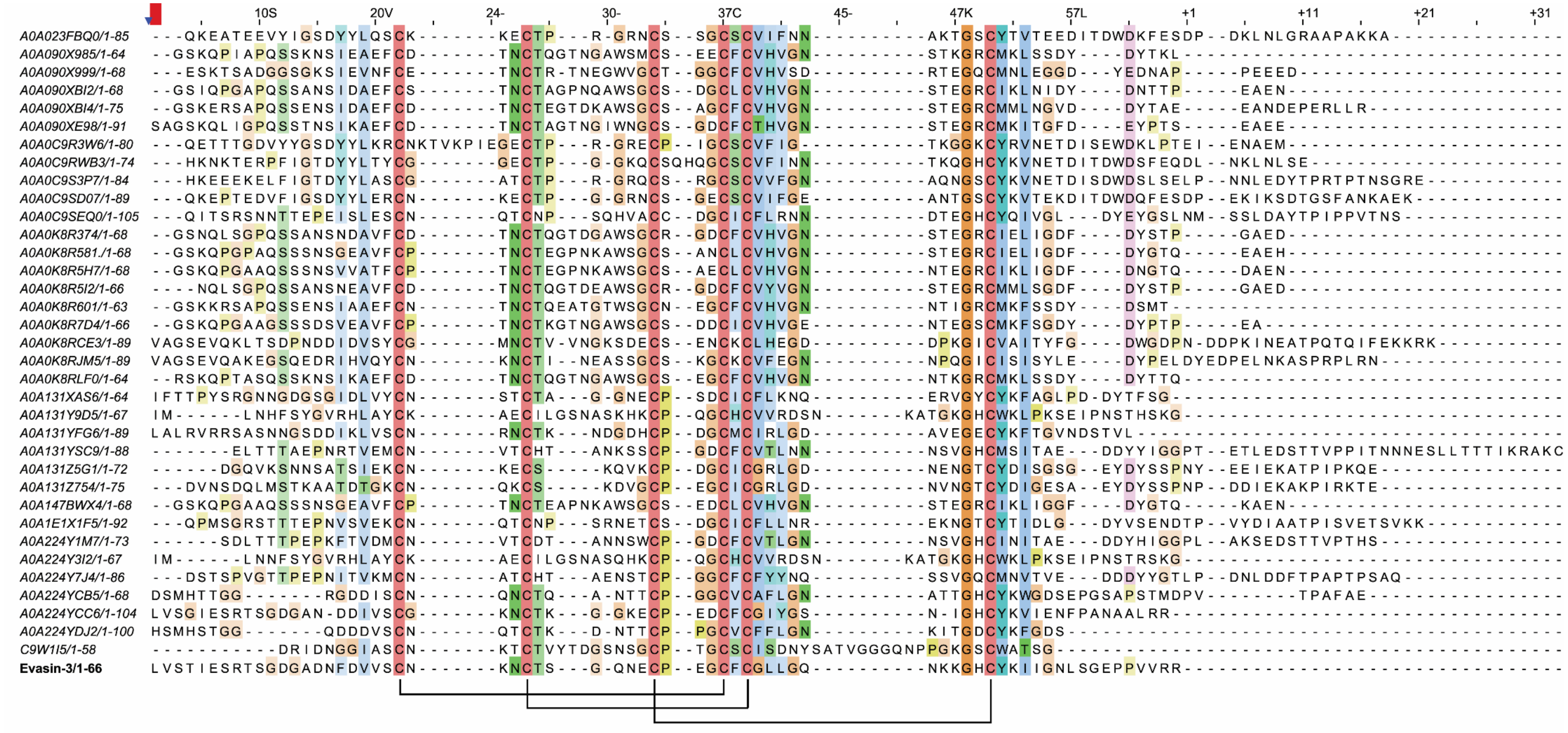

Figure S13. Multiple sequence alignment of identified Evasin-3 homologues. Evasin-3 sequence is set as a reference; disulphide connectivity is schematically represented by lines.

HADDOCK score	-92.9 +/- 1.6
Cluster size	34
RMSD from the overall lowest-energy structure, Å	0.8 +/- 0.5
Van der Waals energy	-56.2 +/- 4.1
Electrostatic energy	-146.0 +/- 33.8
Desolvation energy	-12.4 +/- 4.9
Restraints violation energy	49.7 +/- 28.07
Buried Surface Area	1612.9 +/- 103.3
Z-Score	Z-Score

Table S1. HADDOCK report for the top cluster of the docked CXCL8/tEv3 17-56 model
